# AZGP1 inhibits soft tissue sarcoma cells invasion and migration

**DOI:** 10.1186/s12885-017-3962-5

**Published:** 2018-01-22

**Authors:** Jiayong Liu, Haibo Han, Zhengfu Fan, Marc El Beaino, Zhiwei Fang, Shu Li, Jiafu Ji

**Affiliations:** 1 Key laboratory of Carcinogenesis and Translational Research (Ministry of Education/Beijing), Department of Bone and Soft Tissue Tumor, Peking University Cancer Hospital & Institute, 52 Fucheng Rd., Beijing, 100142 People’s Republic of China; 2 Key laboratory of Carcinogenesis and Translational Research (Ministry of Education/Beijing), Department of Biobank, Peking University Cancer Hospital & Institute, 52 Fucheng Rd., Beijing, 100142 People’s Republic of China; 3 Department of Orthopedic Oncology, MD Anderson Cancer Center, Unit 1448, 1515 Holcombe Boulevard, Houston, Texas 77030 USA; 4 Key laboratory of Carcinogenesis and Translational Research (Ministry of Education/Beijing), Division of Gastrointestinal Cancer Translational Research Laboratory, Peking University Cancer Hospital & Institute, 52 Fucheng Rd., Beijing, 100142 People’s Republic of China; 5 Department of Gastrointestinal Surgery, Peking University Cancer Hospital & Institute, 52 Fucheng Rd., Beijing, 100142 People’s Republic of China

**Keywords:** AZGP1, Soft tissue sarcoma, Metastasis, Survival, Invasion, Migration

## Abstract

**Background:**

One of the major challenges in soft tissue sarcomas is to identify factors that predict metastasis. AZGP1 is a potential biomarker of cancer progression, but its value in soft tissue sarcomas remains unknown. The aim of this study is to determine the expression level of AZGP1 in soft tissue sarcomas, and to analyze its influence on tumor progression.

**Methods:**

AZGP1 immunohistochemistry (IHC) and RT-PCR were performed in 86 patients with soft tissue sarcomas. The relationships between AZGP1 levels and clinicopathologic features were analyzed. In vitro experiments were performed using fibrosarcoma (HT1080), rhabdomyosarcoma (RD) and synovial sarcoma (SW982) cell lines to corroborate our findings. We used lentiviral over-expression and knockdown assays to examine how changes of AZGP1 expressions might affect cellular migration and invasion.

**Results:**

The quantitative RT-PCR results showed that AZGP1 expression was negatively correlated with metastasis and overall survival in soft tissue sarcomas (*p* < 0.05). Immunohistochemical staining showed lower expression of AZGP1 in patients with metastasis than in those without. Kaplan-Meier survival analysis showed that patients with low expression of AZGP1 had shorter overall (*p* = 0.056) and metastasis-free survivals (*p* = 0.038). These findings were corroborated by our in vitro experiments. Over-expression of AZGP1 significantly decreased RD cellular migration and invasion by 64% and 78%, respectively. HT1080 cells migration was inhibited by 2-fold, whereas their invasion was repressed by 7-fold after AZGP1 knockdown.

**Conclusions:**

Our study reveals that reduced AZGP1 expression correlates with in vitro cellular migration and invasion. In vivo, it is associated with higher metastatic risk and shorter survival in patients with soft tissue sarcomas.

**Electronic supplementary material:**

The online version of this article (10.1186/s12885-017-3962-5) contains supplementary material, which is available to authorized users.

## Background

About 40% of individuals with intermediate- or high-grade soft-tissue sarcoma (STS) experience distant relapse, which directly determines the prognosis and affects the therapeutic strategy in these diseases [1, 2]. No gene has been, however, consistently found to be associated with metastasis or to influence prognosis in these tumors. Despite being a major challenge, the identification of such biomarker might be of both prognostic and therapeutic value in STSs.

GEO (Gene Expression Omnibus) is a public repository for high-throughput screening of potential candidate genes associated with tumorgenesis and metastasis. Here, we analyzed the GDS2736 data consisting of 105 samples including 3 cases of lipoma, 3 cases of well differentiated liposarcoma and 99 cases of other types of sarcomas. We identified three candidate genes correlated with sarcoma malignancy, including LOXL2, PARP1 and AZGP1. We then measured the expression of these genes in 81 cases of sarcoma samples by Q-PCR, and analyzed the relationship between their expression and tumor metastasis and overall survival. Of the three candidates, AZGP1 was found to be significantly associated with metastasis and 4-year overall survival.

Zinc alpha2 glycoprotein (AZGP1) was initially found to be associated with lipid degeneration in cachexia and obesity [3, 4]. Recent studies have demonstrated a metastatic and prognostic role of this protein in several malignancies, including prostate, breast, lung, colorectum and liver carcinomas [5–12]. Nevertheless, no studies have been undertaken to evaluate the value of AZGP1 in STSs.

The aim of the current study was to measure, by RT-PCR and IHC, the levels of AZGP1 in STS samples, and to detect their association with prognosis and metastasis in such diseases. We also investigated the effect(s) of AZGP1 under- or over-expression on cellular invasion and migration of some STS cell lines to corroborate our findings.

## Methods

**Fig. 1:**
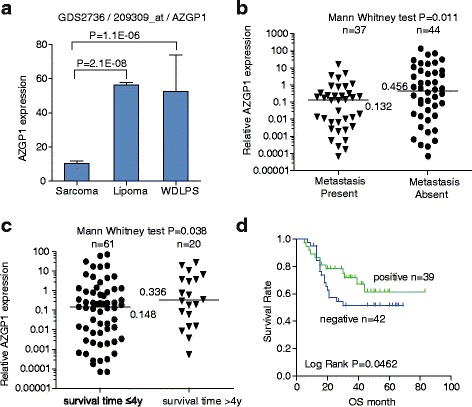
Down-regulation of AZGP1 mRNA was associated with metastases in STS specimens. **a** AZGP1 expression obtained from Gene Expression Omnibus (GEO) database of on Pubmed (GDS2736) was analyzed. **b** and **c** qPCR analysis of AZGP1 expression in 81 cases of STS specimens. **d** Kaplan-Meier curves for the overall survival (OS) of patients were compared between groups with high and low levels of AZGP1. Horizon lines in (**b** and **c**) indicate the median values for each group

Prior to this study, we downloaded the GEO database from Pubmed for analysis of the relation between AZGP1 and metastasis in sarcomas. Involving 105 STS microarray analysis, GDS2736 indicated that AZGP1 expression in sarcomas with high metastatic potential was significantly lower than that in lipoma and well-differentiated liposarcoma (WDLPS) with no or rare metastasis (Fig. 1a).

### Patients and tissue specimens

Tumor samples from 86 patients with primary STS, who underwent surgery between 2007 and 2014, were obtained from the tissue bank of our institution and snap-frozen in liquid nitrogen immediately after surgical resection until RNA extraction. Paraffin-embedded specimens were used for IHC staining. To be included in our study, cases should be histologically diagnosed as grade 2 or 3 according to the FNCLCC grading system, with no preoperative chemotherapy. Metastasis, overall (OS), 4-year, metastasis-free (MFS), as well as disease-specific (DSS) survivals, were monitored with a mean follow-up of 45 months (range 23–83 months). Sample acquisition was approved by the Ethics Committee of the Hospital. Written informed consent was obtained from all patients.

### RNA extraction and quantitative RT-PCR

Quantitative RT-PCR was used to detect the expression levels of AZGP1 mRNA in 81 cases of tumor tissues, since 5 cases were excluded due to RNA extraction failure. Total RNA was extracted from frozen tissues containing > 80% STS cells by RNA Extraction Kit (QIAgen) according to the manufacturer’s instructions. Quantity and quality of RNA was confirmed by a NanoDrop 2000 Spectrophotometer (Thermo Fisher Scientific, Wilmington, DE, USA). RNA purity was determined by an OD260/280 value between 1.8 and 2.0. For mRNA expression, cDNA was obtained from 2 μg total RNA using Moloney murine leukemia virus reverse transcriptase (M-MLV RT) (Invitrogen, Carlsbad, CA) with oligodT_15_ primers. GAPDH mRNA was used as an endogenous control to normalize for AZGP1 mRNA expression. qRT-PCR was performed using SYBR® Green PCR Master Mix (TOYOBO) on the ABI 7500 Fast (Applied Biosystems). Data were calculated as relative quantification to GAPDH, based on calculations of 2^−△Ct^ where −△Ct = Ct (Target) – Ct (Reference). Fold change was presented by the 2^−△△Ct^ method [13]. Sequences of all primers are listed on Additional file 1: Table S1.

### Immunohistochemistry

Immunohistochemical staining for AZGP1 was performed in soft tissue sarcoma tissue microarray (TMA) by a standard two-step method. Briefly, the TMA sections were dried overnight at 37 °C, deparaffinized in xylene and rehydrated through a series of graded alcohol. Endogenous peroxidase activity was blocked with 3% hydrogen peroxide for 20 min. The slides were boiled in 10 mM sodium citrate buffer pH 6.0 by a pressure cooker for 10 min. After washing three times with phosphate buffered saline (PBS; 0.01 mol/L; pH = 7.4), the slides were incubated with 5% non-fat milk in PBS for 30 min to reduce nonspecific antibody binding. Subsequently, slides were incubated overnight at 4 °C with the rabbit polyclonal antibody against human AZGP1 (Abcam; Cambridge, UK; 1:100 dilution). After rinsing, the slides were incubated with goat anti-rabbit antibody (Jackson ImmunoResearch Laboratories, West Grove, PA) at a 1:100 dilution in PBS for 1 h at room temperature, and stained with 3,3-diaminobenzidine tetrahydrochloride (DAB). Finally, they were counterstained with Mayer’s hematoxylin, dehydrated in graded alcohols followed by xylene. Known immunostaining-positive specimens were used as positive controls and slides immunoreacted with PBS were used as the negative controls.

### Western blotting

Total protein from cells was extracted in RIPA lysis buffer and quantified using BCA assay. 20 μg protein from each sample was separated by 10% SDS polyacrylamide gel electrophoresis and electroblotted onto polyvinylidene difluoride (PVDF) membranes (Millipore, Bedford, MA). The membranes were blocked in 5% non-fat milk for 1 h, washed three times with Tris-buffered saline containing 1% Tween 20 (TBST) at room temperature and then incubated overnight at 4 °C with the rabbit polyclonal antibody against human AZGP1 (Abcam; Cambridge, UK; 1:2000 dilution). After washing with TBST, membranes were incubated with secondary antibodies at room temperature for 1 h (goat anti-rabbit IgG, 1:10,000 dilution, Jackson ImmunoResearch Laboratories). Following washing with TBST, immunoreactivity was visualized by enhanced chemiluminescence reagents (Millipore). GAPDH served as internal reference.

### Cell culture

Three human STS cell lines (RD ATCC^®^ Number: HTB-166™, SW982 ATCC^®^ Number: HTB-93™ and HT1080 ATCC® Number: CCL-121™) were purchased from American Type Culture Collection. RD and HT1080 cells were cultured in RPMI-1640 medium (Gibco, CA, USA) containing 10% fetal bovine serum (FBS; Gibco) at 37 °C with a humidified 5% CO2 atmosphere. SW982 cells were cultured in Leibovitz’s L-15 medium (Gibco, CA, USA) containing 10% FBS.

### Vector construction

All constructs were made by standard DNA recombination techniques. The human AZGP1 (NM_001185.3) sequences were amplified by PCR from cDNA using primers listed in Additional file 1: Table S1, and subsequently cloned into lentiviral shuttle vector plenti6 (Invitrogen). For AZGP1 knockdown constructs, two short hairpin RNA (shRNA) sequences, including shRNA150 (target sequence: 5’-GGCTCACTCAATGACCTCCAG-3′), shRNA368 (target sequence: 5’-GTGAGATCGAGAATAACAGAA-3′) and scramble control sequence of 5’-GCTTCGCGCCGTAGTCTTA-3′ were designed and cloned into lentiviral shuttle plenti6-U6 vector.

### Cell transfection

Lentiviral constructs were transfected into human HEK 293 T cells (ATCC^®^ Number: CRL-11268™) with the ViraPower Packaging Mix (Invitrogen) to generate lentivirus. For infection, RD cells were seeded into 6-well plates at a density of 5 × 10^4^ cells/well, and infected with AZGP1 over-expression lentivirus or empty lentivirus as control. HT1080 cells were infected with shRNA lentivirus or scramble lentivirus as control. Antibiotic-resistance cells were selected by 5 μg ml^−1^ blasticidin (Invitrogen) and used for subsequent experiment.

### Wound healing assay

Cell spreading was analyzed using the wound healing assay. RD cell layers at 90% density in 24-well plates were scratched with a sterile 200 μL pipette tip and then washed with PBS. After 48 h, spreading cells were observed under the microphotography. Assays were repeated three times for each clone.

### Transwell migration and invasion assay

Cell migration or invasion assay was performed in a 24-well Boyden chamber with or without Matrigel as described elsewhere [14]. The cells on the lower surface of the membrane were stained with crystal violet after fixation with 2% methanol for 5 min. Photographs of four randomly selected fields were taken to indicate cells that migrated to the other side of the membrane, and cell numbers were counted under a microscope at 200× magnification. Each test was performed in triplicate.

### Statistical analysis

We calculated OS, 4-year, MFS and DSS using Kaplan-Meier analysis. We defined OS as the period between the date of the definitive surgery and the date of death, the 4-year survival as the period between the date of the definitive surgery and 4 years after, MFS as the interval between the date of the definitive surgery and the appearance of metastasis, and DSS as the date between the date of the definitive surgery and the time of death resulting from the disease itself. The effect of AZGP1 expression on Kaplan Meier survival curves was evaluated by the Log Rank test.

Mann Whitney and Kruskal Wallis tests were used to detect any association between AZGP1 mRNA expression and various pathological features (gender, age, TNM classification, recurrence, metastasis, and 4-year survival) between 2 or more groups, respectively.

Univariate analysis between pathological features (age, gender, AZGP1 expression, tumor size and histological grade) and metastasis or disease-specific survival was determined using Pearson’s correlation analysis. Multivariate analysis between the same variables was evaluated by the Cox regression model. Unpaired student’s T-test was performed to evaluate cell migration/invasion after gene modulation. All analyses were performed with SPSS^®^ software 23.0 program for Windows^®^ (SPSS Inc., Chicago, IL, USA). The statistical significance between groups was set at a *p*-value < 0.05.

## Results

### Patients with low AZGP1 expression had more metastasis and less 4-year survival

qRT-PCR results showed that levels of AZGP1 in patients with metastasis were 4 times lower than in those without (Fig. 1b and Table 1, median value 0.132 vs. 0.456, *p* = 0.0113). The levels of AZGP1 in patients with low 4 years’ survival were 2 times lower than in those who lived more than 4 years (Fig. 1c and Table 1, median value 0.148 vs. 0.336, *p* = 0.038).

**Table 1:** Univariate correlation between AZGP1 mRNA expression and pathological features in STS patients

Variable		Case no.	AZGP1 expression (RQ: 2^-△Ct^)	*P-*value^a^
			Median	
*Gender*	Male	51	0.2271	0.3790
	Female	30	0.1890	
*Age* (year)	≤ 60	51	0.2322	0.3679
	> 60	30	0.1400	
*TNM*	II	35	0.1905	0.7081
	III	46	0.2069	
*Recurrence*	Absent	51	0.2000	0.8125
	Present	30	0.2000	
*Metastasis*	Absent	44	0.4560	0.0113
	Present	37	0.1320	
*Survival* (year)	< 4	61	0.1480	0.0377
	≥ 4	20	0.3355	

^a^Mann–Whitney test for two groups; Kruskal-Wallis test for more than two groups

### Patients with low expression of AZGP1 had shorter overall survival

According to the median value of AZGP1 expression (0.2014) in STS samples, patients were divided into low and high expression groups. Kaplan-Meier survival analysis showed that patients with low AZGP1 expression had significantly shorter overall survival (OS) than those with high expression (Fig. 1d, 75th percentile was 16 vs. 30 months, *p* < 0.05).

### AZGP1 expression was related to metastasis and disease-specific mortality

**Fig. 2:**
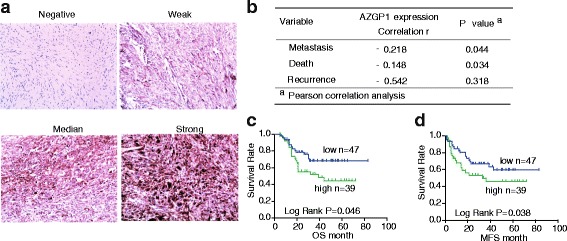
Down-regulation of AZGP1 protein was associated with metastases and short survival in STS specimens. **a** AZGP1 expression levels in STS specimens were determined by IHC staining. **b** Pearson’s correlation analysis of AZGP1 expression levels and metastasis and disease-specific death. **c** and **d** Kaplan-Meier curves for the overall survival (OS) and metastasis-free survivals (MFS) of patients were compared between groups with high and low levels of AZGP1 protein

To further analyze the relationship between AZGP1 level and metastasis or survival of STS patients, we performed IHC staining analysis in TMA with 86 cases of STS tissue samples (Fig. 2a). Patients were divided into low (negative and weak expression) and high expression groups (median and strong expression). Pearson’s correlation analysis showed that AZGP1 expression negatively correlates with STS metastasis and disease-specific mortality (Fig. 2b; *r* = −0.218, *p* = 0.044; *r* = −0.148, *p* = 0.034, respectively). Consistent with our findings, univariate analysis showed a higher average hazard ratio (HR) for metastasis and disease-specific mortality (Table 2; HR = 3.731, *p* < 0.05; HR = 2.481, p < 0.05, respectively) in patients with low AZGP1 expression. The results suggest that patients with low expression of AZGP1 are more prone to metastasis and disease-specific death.

**Table 2:** Univariate analysis between metastasis or death and pathological features in STS patients

Variables	Metastasis		*P* Value	Death		*P* Value
	HR	95%CI		HR	95%CI	
Age	1.339	0.545–3.289	0.524	0.725	0.289–1.821	0.494
(≥ 60 yr. vs. < 60 yr)						
Gender	0.711	0.287–1.762	0.461	0.902	0.362–2.248	0.825
(Male vs. Female)						
Tumor size	2.000	0.786–5.088	0.146	3.286	1.202–8.982	0.020
(> 5 cm vs. ≤ 5 cm)						
Histological grade	3.750	1.493–9.420	0.005	1.086	1.362–8.712	0.009
(G3 vs. G2)						
AZGP1 expression	3.731	1.770–10.204	0.035	2.481	1.022–6.024	0.044
(low vs. high)						

Kaplan-Meier survival analysis suggested that patients with low AZGP1 expression exhibited significantly shorter OS and MFS than those with high expression (Fig. 2c and d, 75th percentile was 15 vs. 30 months for OS, *p* = 0.046; 6 vs. 18 months for MFS, *p* = 0.038). Multivariate survival analysis using Cox’s regression model, however, failed to identify AZGP1 expression as an independent prognostic factor (data not shown). Consistent with our qRT-PCR results, these data also suggested that low expression of AZGP1 protein were correlated with metastasis and short survival.

### AZGP1 expression in STS cells

**Fig. 3:**
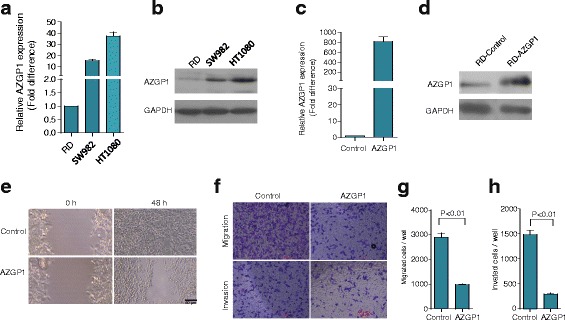
Ectopic expression of AZGP1 inhibited RD cell spreading, migration and invasion. **a** and **b** The expression level of AZGP1 in STS cell lines were determined by qPCR and western blot. **c** and **d** qPCR and western blot analysis were performed to confirm ectopic expression of AZGP1 in RD cells. **e** Wound healing assay showed the spreading of cells was significantly retarded after AZGP1 over-expression compared with control cells. **f** Boyden chamber assays showed that cell migration and invasion through matrigel were remarkably suppressed in AZGP1 over-expressing cells compared with the control cells, respectively. The quantification results of migrated cells and invaded cells through matrigel are plotted in (**g** and **h**), respectively. Data in (**g** and **h**) represent the mean ± SD from three independent experiments

We then analyzed AZGP1 expression in three STS cell lines (RD, SW982 and HT1080) by qRT-PCR and Western blot. The results showed that AZGP1 mRNA and protein levels in RD cells were lower than those in HT1080 and SW982 cells (Fig. 3a and b).

### AZGP1 inhibited cell spreading, migration and invasion in RD cells

In order to document the effects of AZGP1 on cell movement, we tested the cellular spreading capability by the wound healing assay, and the cell migration and invasion ability by Transwell assay after ectopic expression of AZGP1 in RD cells. As shown in Fig. 3c and d, the expression of AZGP1 was up-regulated significantly after RD cells infection with AZGP1 lentivirus. Following the increase in AZGP1 levels, RD cell spreading decreased compared to that of control cells (Fig. 3e). The migration and invasion of cells over-expressing AZGP1 were also decreased by 62% and 81% respectively, compared with control cells (Fig. 3f–h). These results suggested that AZGP1 over-expression had an inhibitory effect on cell spreading, migration and invasion in RD cells.

### AZGP1 inhibition promoted migration and invasion in HT1080 cells

**Fig. 4:**
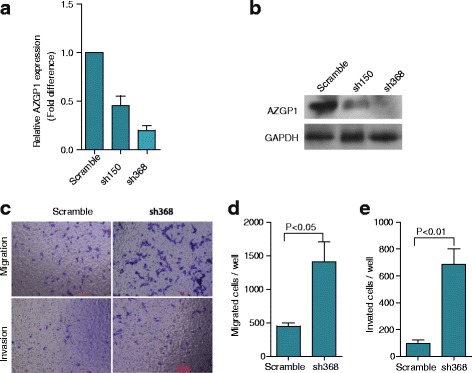
Inhibition of AZGP1 increased HT1080 cell migration and invasion. **a** and **b** The expression of AZGP1 was suppressed after transfecting shRNA lentivirus into HT1080 cells compared with the scramble control cells. **c** Boyden chamber assays showed that cell migration and invasion through matrigel were remarkably increased in AZGP1-sh368 inhibited cells compared with the control cells, respectively. The quantification results of migrated cells and invaded cells through matrigel are plotted in (**d** and **e**). Data in (**d** and **e**) represent the mean ± SD of three independent experiments

We inhibited the expression of AZGP1 using small hairpin RNA (shRNA) in HT1080 cells. As shown in Fig. 4a and b, the expression of AZGP1 mRNA and protein was decreased by 55% for sh150 and 80% for sh368 compared with the control (scramble oligo). As demonstrated by Transwell assay (Fig. 4c), the number of migrated cells was increased by 3.1 fold (Fig. 4d), and the number of invasive cells was enhanced by 5.2 times (Fig. 4e) after knockdown of AZGP1 expression in HT1080 cells by sh368 lentivirus. These findings were in accordance with those inaugurated from the RD cells experiments, and suggested that AZGP1 inhibition promoted cell migration and invasion.

## Discussion

Our in vivo and in vitro results suggest that AZGP1 has a prognostic and a metastatic value in STSs. It has been reported that patients with prostate or lung cancer with low AZGP1 levels had worse survival compared to those with high levels [13, 15]. Similarly, absent or weak expression of AZGP1 was found to be associated with recurrence and metastasis of prostate cancer patients [10, 12, 16]. In their recent tissue microarray analysis on 11,152 samples, Burdelski et al. showed that the prognostic value of AZGP1 was comparable to the strongest established prognostic biomarkers in prostate cancers [12]. To our knowledge, our study is the first to assess AZGP1 expression levels in STS patients. Consistent with the available literature in other cancer subtypes, both qRT-PCR and IHC analyses demonstrated that STS patients with metastasis or shorter overall survival had lower AZGP1 levels. Additionally, univariate analysis indicated that reduced AZGP1 expression was a risk factor for metastasis and disease-specific death. AZGP1 over-expression experiments significantly decreased STS cell lines migration and invasion, corroborating our in vivo results. Such finding has a high clinical impact, since no gene or factor has been consistently found to predict prognosis in these diseases. Although we didn’t evaluate the expression of AZGP1 in normal tissues, data from GSD1282 supported our results. We analyzed the expression level of AZGP1 in fetal kidney control samples (FK) and clear cell sarcoma of the kidney (CCSK). Compared with FK tissues, the expression of AZGP1 in CCSK tissues was remarkably decreased (median expression, 231.4 vs. 86.2, *p* = 0.02). Together, these results suggest that AZGP1 is a potential prognostic marker for STS. Since multivariate analysis failed to detect such a relationship, further investigations with larger sample size are needed to validate our findings.

AZGP1 had been known as a protein involved in the control of fat degradation and energy expenditure [4, 17]. It remains unclear, however, how it participates in the process of cancer development and progression. In recent years, there were more evidence of a close relationship between lipid metabolism and tumor progression. Increased lipogenesis has been reported to be associated with poor prognosis in breast, prostate, and colon cancer [18–20]. As for sarcomas, Patel et al. found that lipid droplets accumulate in human malignant peripheral nerve sheath tumor (MPNST) cell lines and primary human tumors. Inhibition of fatty acid synthesis, which is overexpressed in MPNST cells lines, can effectively reduce MPNST survival and delay tumor growth in vivo [21]. Therefore, decreased AZGP1 expression, which was found to be more prevalent in metastatic patients in our study, may increase the process of lipogenesis and affect prognosis of STS patients.

Due to the high homology between AZGP1 and MHC class I sequence and structure, AZGP1 might be involved in tumor proliferation and invasion by different mechanisms. By binding hydrolases, it activates apoptosis and suppresses tumor invasion [22]. It also inhibits tumor cell proliferation by down-regulating cyclin-dependent kinase 1, leading to G2/M phase arrest [23]. AZGP1 might also inhibit tumor invasion by suppressing TGF-β-mediated epithelial-to-mesenchymal transition, which plays a critical role in cancer progression [24, 25]. Chang et al. reported that AZGP1 may inhibit tumor growth and metastasis by blocking the mTOR pathway [14, 26]. In addition, AZGP1 expression was linked with PTEN deletion and might regulate the phosphatidylinositol-3 kinase (PI3K/AKT) pathway, which can trigger a cascade of responses to drive cell proliferation and tumor progression [27]. Apart from the aforementioned properties, there is accumulating evidence to suggest that AZGP1 modulates cell attachment and spreading [28]. Likewise, our results show that AZGP1 over-expression inhibits RD cell spreading, migration and invasion, while its inhibition decreased HT1080 cell migration and invasion. The exact mechanism for AZGP1-mediated antineoplastic properties in STSs remains however uncertain, and, hence, requires further investigations.

## Conclusions

In summary, our study demonstrated that low AZGP1 expression was associated with higher invasive and metastatic cellular potentials in soft-tissue sarcomas. AZGP1 might constitute a potential prognostic biomarker and therapeutic target in such diseases. Further studies are needed to validate this finding.

## Additional file

Additional file 1: Table S1. Primers sequence. The primer names and sequence for Q-PCR analysis of ZAG and recombinant plasmid construct were listed in the table. (DOC 34 kb)

## Abbreviations

AZGP1: Zinc-alpha2-glycoprotein
BCA: Bicinchoninic acid
cDNA: Complementary deoxyribonucleic acid
DAB: 3, 3-diaminobenzidine tetrahydrochloride
FNCLCC: French Federation of Cancer Centers Sarcoma
GAPDH: Glyceraldehyde-3-phosphate dehydrogenase
GEO: Gene Expression Omnibus
HR: Hazard ratio
IHC: Immunohistochemistry
MFS: Metastasis free survival
MHC: Major histocompatibility complex
M-MLV RT: Moloney murine leukemia virus reverse transcriptase
mRNA: Messenger ribonucleic acid
mTOR: Mechanistic target of rapamycin
OS: Overall survival
PBS: Phosphate buffered saline
PCR: Polymerase chain reaction
PVDF: Polyvinylidene difluoride
RD: Rhabdomyosarcoma
RNA: Ribonucleic acid
RT-PCR: Real time-polymerase chain reaction
SD: Standard deviation
SDS: Sodium dodecyl sulfate
shRNA: Small hairpin RNA
STS: Soft tissue sarcoma
TBST: Tris Buffered Saline with Tween 20
TGF: Transforming growth factor
TMA: Tissue microarray
WDLPS: Well-differentiated liposarcoma
