# Supplementary material for: AZGP1 inhibits soft tissue sarcoma cells invasion and migration
Source: BMC Cancer. 2018 Jan 22;18:89. doi: 10.1186/s12885-017-3962-5 (PMC5778744; doi:10.1186/s12885-017-3962-5)
Supplement: Additional file 1: Table S1. — Primers sequence. The primer names and sequence for Q-PCR analysis of ZAG and recombinant plasmid construct were listed in the table. (DOC 34 kb) [file 12885_2017_3962_MOESM1_ESM.doc]

Table S1. Primers sequences.

| ***Primer name Sequence*** | |
| --- | --- |
| ***Q-PCR analysis of ZAG*** | |
| ZAG forward primer | 5′-GGAGACCCTGAAAGACATCG-3′ |
| ZAG reverse primer | 5′-TTGGTTATCTGGGCTGCTGG-3′ |
| GAPDH forward primer | 5′-GACCCCTTCATTGACCTCAAC-3′ |
| GAPDH reverse primer | 5′-CTTCTCCATGGTGGTGAAGA-3′ |
|  |  |
| ***Recombinant plasmid construct*** | |
| AZGP1-CDS forward primer | 5′-CGGGATCCATGGTAAGAATGGTGCCTGTCCTG-3′ |
| AZGP1-CDS reverse primer | 5′-CCGCTCGAGCTAGCTGGCCTCCCAGGGCACCA-3′ |
| AZGP1-shRNA-150-sense | 5′-GATCCCGGCTCACTCAATGACCTCCAGTTCAAGAGACTGGAGGTCATTGAGTGAGCCTTTTTC-3′ |
| AZGP1-shRNA-150-antisense | 5′-TCGAGAAAAAGGCTCACTCAATGACCTCCAGTCTCTTGAACTGGAGGTCATTGAGTGAGCCGG-3′ |
| AZGP1-shRNA-368-sense | 5′-GATCCCGTGAGATCGAGAATAACAGAATTCAAGAGATTCTGTTATTCTCGATCTCACTTTTTC-3′ |
| AZGP1-shRNA-368-antisense | 5′-TCGAGAAAAAGTGAGATCGAGAATAACAGAATCTCTTGAATTCTGTTATTCTCGATCTCACGG-3′ |
